# Superplastic Deformation Mechanisms of Superfine/Nanocrystalline Duplex PM-TiAl-Based Alloy

**DOI:** 10.3390/ma10091103

**Published:** 2017-09-19

**Authors:** Xuebo Gong, Zhenxin Duan, Wen Pei, Hua Chen

**Affiliations:** School of Materials Science and Engineering, Changchun University of Technology, Changchun 130012, China; 15948334230@163.com (X.G.); 18844061816@163.com (Z.D.); peiwen19941102@163.com (W.P.)

**Keywords:** duplex PM-TiAl alloy, superfine/nanocrystalline, superplastic deformation

## Abstract

In this paper, the equiaxed superfine/nanocrystalline duplex PM-TiAl-based alloy with (γ + α_2_) microstructure, Ti-45Al-5Nb (at %), has been synthesized by high-energy ball milling and vacuum hot pressing sintering. Superplastic deformation behavior has been investigated at 1000 °C and 1050 °C with strain rates from 5 × 10^−5^ s^−1^ to 1 × 10^−3^ s^−1^. The effects of deformation on the microstructure and mechanical behaviors of high Nb containing TiAl alloy have been characterized and analyzed. The results showed that, the ultimate tensile strength of the alloy was 58.7 MPa at 1000 °C and 10.5 MPa at 1050 °C with a strain rate of 5 × 10^−5^ s^−1^, while the elongation was 121% and 233%, respectively. The alloy exhibited superplastic elongation at 1000 and 1050 °C with an exponent (m) of 0.48 and 0.45. The main softening mechanism was dynamic recrystallization of γ grains; the dislocation slip and γ/γ interface twinning were responsible for superplastic deformation. The orientation relationship of γ/γ interface twinning obeyed the classical one: (001)_γ_//(110)_γ_.

## 1. Introduction

TiAl-based alloys (γ-TiAl + α_2_-Ti_3_Al duplex alloys) have been regarded as a potential light-weight material candidate for advanced structural applications due to its excellent properties, such as low density, high specific strength and stiffness, and sufficient oxidation resistance [1,2,3,4]. However, the intrinsic brittleness of TiAl-based alloys make it difficult to treat by conventional processing. Therefore, the superplasticity of TiAl-based alloys have received much attention. The superplastic forming is the most effective technology to produce the near-net-shape and complex structural components [5,6]. In order to further improve the mechanical properties and the potential to remarkably increase the service temperature range of TiAl alloys, which had been investigated in many studies [7,8,9,10].

Gerling [11] investigated the structural characterization and tensile properties of Ti-46Al-9Nb sheet from 700 to 1000 °C at an initial strain rate of 10^−4^ s^−1^. Cheng et al. [12] found that the microstructure evolution was characterized by grain refinement and an increasing fraction of low-angle grain boundaries. Li et al. [13] found different mechanisms have been proposed to explain the superplastic deformation of TiAl-based alloys, which is considered as the combination of dislocation movements, mechanical twinning, and dynamic recrystallization. Many investigations have been conducted to study that the addition of Nb to the alloys, which might slow the domain growth, promote non-basal slip, restrain planar slip, and even reduce antiphase boundary (APB) energy [14,15].

Superplasticity generally features equiaxed and fine-grained microstructures. Recent studies have exploited coarse, as-cast microstructures in the preparation of fine-grained microstructures and, eventually, superplastic materials. The major drawback of this approach is that it limits the shapes of the materials produced. We have used a number of new TiAl intermetallic compounds in the preparation of superplastics. It is widely accepted that a fine-grained microstructure is a prerequisite for superplasticity. Unfortunately, the high melting point of TiAl intermetallic compounds renders it difficult to achieve an ultra-fine grain structure for them. Therefore, such efforts are confined to the low melting α, β, or α + β forms of the intermetallic compounds [16,17].

However, a series of investigations focused on the micron-scale structure, and the superfine/nanocrystalline duplex (γ + α_2_) microstructures of the PM-TiAl-based alloys are seldom reported. Therefore, it is necessary to investigate the tensile deformation behavior of superfine duplex TiAl-based alloys at elevated temperature (near 1000 °C) with relatively low strain rates.

The purpose of the present work is to investigate superplastic deformation of PM-TiAl-based alloys at 1000 °C and 1050 °C with strain rates from 5 × 10^−5^ s^−1^ to 1 × 10^−3^ s^−1^. In addition, high-temperature deformation mechanisms and superplastic deformation behaviors were also analyzed in detail at 1000 °C with 5 × 10^−5^ s^−1^. In order to refine grains, the duplex (γ + α_2_) microstructure of equiaxed superfine/nanocrystalline alloys were synthesized by high-energy ball milling and vacuum hot pressing sintering. Since the microstructural refinement can be effectively achieved by subsequent treatment, an enhanced overall performance of TiAl alloys is expected.

## 2. Experimental Procedures

The nominal composition of the alloy is Ti-45Al-5Nb (at %), and the Ti, Al, and Nb powders (average particle size > 50 μm, purity > 99%) are comminuted into nanometer powder by high-energy ball milling. The mixed powder was subjected to high-energy ball milling for 40 h at a milling speed of 550 r/min and a ball-to-powder weight ratio of 10:1 under an argon atmosphere. Subsequently, the duplex (γ + α_2_) alloy was prepared at 1200 °C for 1 h by ZT-40-20 vacuum hot pressing sintering furnace (pressure 30 MPa). To increase the density of the sintered bulk specimen, it was canned and extruded to a 27% reduction at 1100 °C and 5 × 10^−5^ s^−1^. Tensile samples were cut from the center of the extruded alloy, with a gauge section with a 2 × 2 mm cross-section and 10 mm gauge length. Tensile tests were carried out using an electronic universal testing machine (DDL50, CIMACH, Changchun, China). Deformation temperatures were maintained at 1000 °C and 1050 °C with strain rates of 1 × 10^−3^ s^−1^, 1 × 10^−4^ s^−1^, and 5 × 10^−5^ s^−1^. After the tensile test under an argon atmosphere, the sample was immediately quenched in water to retain the microstructure.

The deformation microstructure and fracture surface were investigated using a scanning electron microscope (SEM, JSM-5500LV, JEOL Ltd., Tokyo, Japan) and laser scanning confocal microscope (LSM-700, ZEISS, Oberkochen, Germany), Furthermore, the mismatch of the structures, orientation relationship of the interface binding, and optimal orientation of the alloy samples after tensile tests were analyzed by transmission electron microscope (TEM, JEM-2000EX, JEOL Ltd., Tokyo, Japan), high-resolution electron microscope (HREM, FEI Talos F20S, FEI Technologies Inc., Beaverton, OR, USA), and electron backscattered diffraction (EBSD, NordlysMax2, Oxford Instruments, Abingdon, UK) operating on a scanning electron microscope (SEM). SEM and TEM observations were performed using a JSM-5500LV and a JEM-2000EX operated at 100 kV, respectively.

## 3. Results and Discussion

### 3.1. The TEM Analysis of Microstructure before and after Canning Extrusion

**Figure 1:**
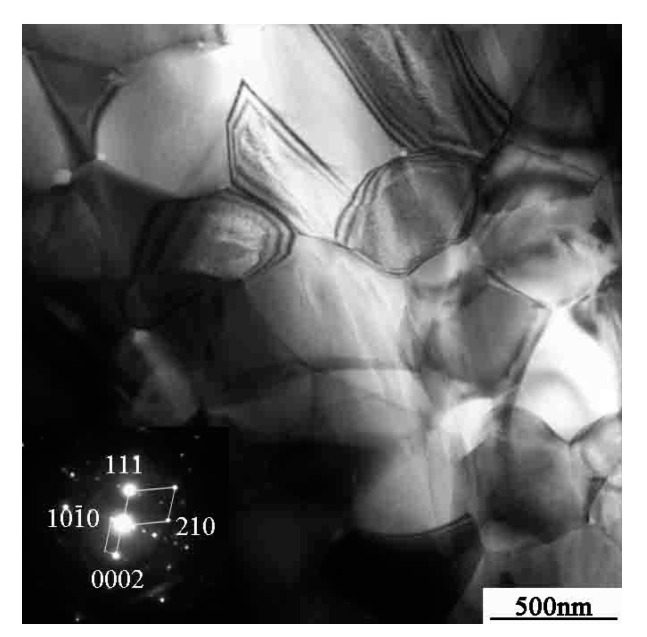
The TEM micrographs of the sintered bulk.

Figure 1 shows the TEM micrographs of the as-sintered bulk specimens. The diffraction pattern analysis shows that the alloy consists of the equiaxed duplex (γ + α_2_) microstructure. The size of the γ phase is 500–800 nm, and the size of the α_2_ phase is about 200 nm. The α_2_ phase is evenly distributed in the matrix of the equiaxed γ phase.

**Figure 2:**
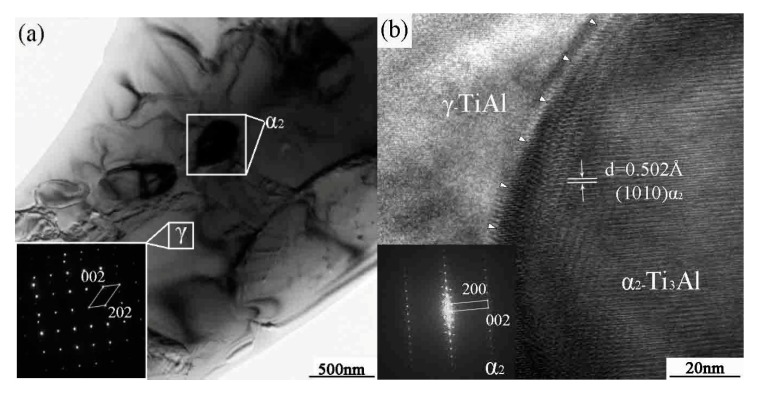
The HREM micrographs of the canning extrusion microstructure. (**a**) TEM micrographs; (**b**) HREM micrographs.

HREM micrographs of the as-extruded microstructure are shown in Figure 2. Compared with the regular polygonal interface of the sintered bulk, the grains have elongated parallel to the extrusion direction. We also observe that the number of grains per unit area increases after the canning extrusion due to the occurrence of dynamic recrystallization. The deformation is uniform, occurring on most of the grains in order to avoid the phenomenon of stress concentration. Meanwhile, the fine grain structure often increases a material’s ability to tolerate plastic deformation without fracture.

### 3.2. True Stress-Strain Curves

**Figure 3:**
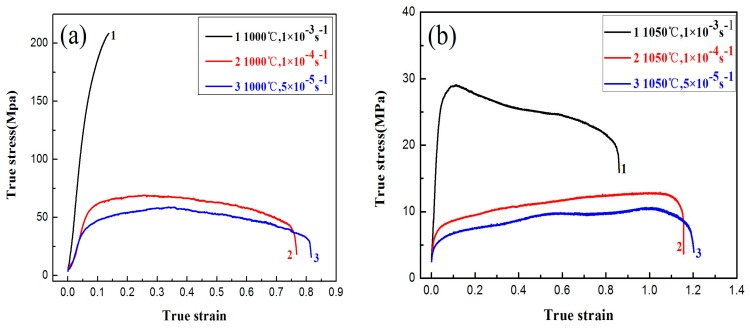
True stress-strain curves of the tensile specimens at different temperature: (**a**) 1000 °C; and (**b**) 1050 °C.

The stress-strain curve reveals the macroscopic response of the deformation mechanism. Figure 3 shows the true stress-strain curves of the tensile specimens at different temperatures and strain rates. The overall trend of the curve is consistent with superplastic behavior. At the initial stage of the tensile test, work hardening is dominant, and the flow stress increases rapidly with the strain. When the stress reaches a peak value, the softening effect begins to be dominated by the acceleration of dynamic recovery and recrystallization, and the stress-strain curves approach a steady state. The peak value of stress decreases with the decrease of the strain rate and the increase of temperature. Meanwhile, the curve appears serrated with repeated oscillation. The main reasons are grain boundary sliding and dynamic recrystallization. When the grain boundary slip is obstructed, the formation of stress concentration causes the flow stress to rise, causing dynamic recrystallization. The increase of the stress also promotes grain boundary sliding. The stress is relaxed and causes the alloy to soften. As the deformation continues, the stress effects strengthen in the new grains, causing stress to rise again (as shown in curve 2 and curve 3 of Figure 3).

**Figure 4:**
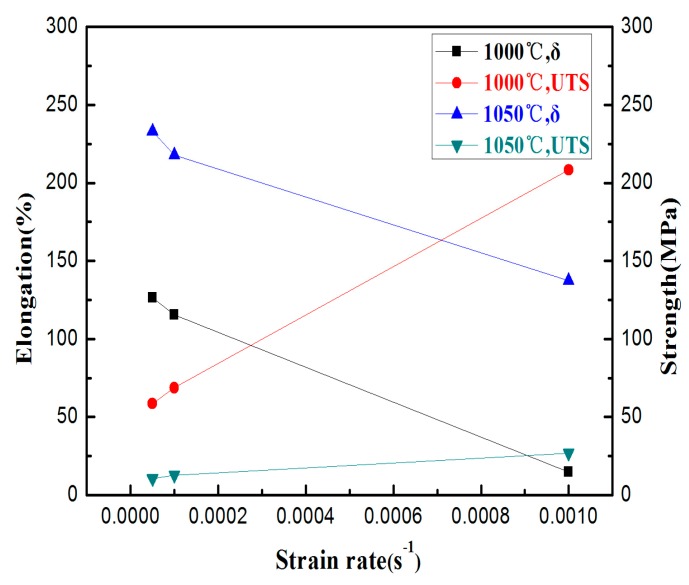
Dependence of tensile properties at different temperatures.

When the strain rate is reduced at constant temperature, a longer steady-state stage is observed with further elongation. As shown in Figure 4, the elongation is 126% at 1000 °C at the strain rate of 5 × 10^−5^ s^−1^. The dislocation density and the increment rate are found to fall with the decrease in strain rate resulting in the deformation of the alloy. When the stress peak is reached, an increase in elongation is observed. The ultimate tensile strength decreases from 58 MPa to 43 MPa when the strain rate decreases from 1 × 10^−4^ s^−1^ to 5 × 10^−5^ s^−1^. The minimum value of the ultimate tensile strength is 10.6 MPa at 1050 °C with a strain rate of 5 × 10^−5^ s^−1^. Compared to the TiAl-based alloy with the micron-scale structure, the ultimate tensile strength of the equiaxed superfine microstructure are significantly lower (as shown in Table 1) [18,19,20]. The ultimate tensile strength is closely related to the strain rate, and the flow stress decreases with the decrease of the strain rate. When the strain rate is lowered, the time required for the deformation process is longer, and the amount of plastic deformation per unit time is reduced. Therefore, the additional time allows dynamic recovery to reduce the dislocation pile-ups and the dislocation pile-ups are relaxed. This leads to a significant reduction in the lattice distortion within the alloy, dislocation slip, and diffusion. Thus, the flow stress is reduced. For the high strain rate of 1 × 10^−3^ s^−1^, the specimen does not enter the steady area that is fractured. The flow stress reached 208 MPa, and the elongation at fracture was 14.5% (as shown in Figure 4). Therefore, the strengthening stage is related to the continuous accumulation of defects and growth of the grains formed in the initial stages. Grain boundary slip causes defects with an increase in strain rates. The rate of formation of the defect is faster than the rate of recovery resulting in a rapid increase in flow stress [21].

The effect of deformation temperature on superplasticity is closely related to the microstructure stability and cavities when the strain rate is constant. In superplastic deformation, the stability and elongation of the microstructure are high. As shown in Figure 4, the elongation of the tensile specimen at 1050 °C is much higher than that at 1000 °C when the strain rate is constant. Meanwhile, the peak value of the alloy’s flow stress decreases with the increase of plastic deformation temperature under the same strain rate condition. Due to the rise in plastic deformation temperature, the atomic energy of the alloy is increased. Therefore, the binding force of the atoms decreases, the thermal vibration amplitude increases, the external thermal activation energy increases, and the activity of atoms is higher. The bonding force of the atoms is weakened, resulting in a significant reduction of the dislocation barrier, and thereby reducing the flow stress.

**Figure 5:**
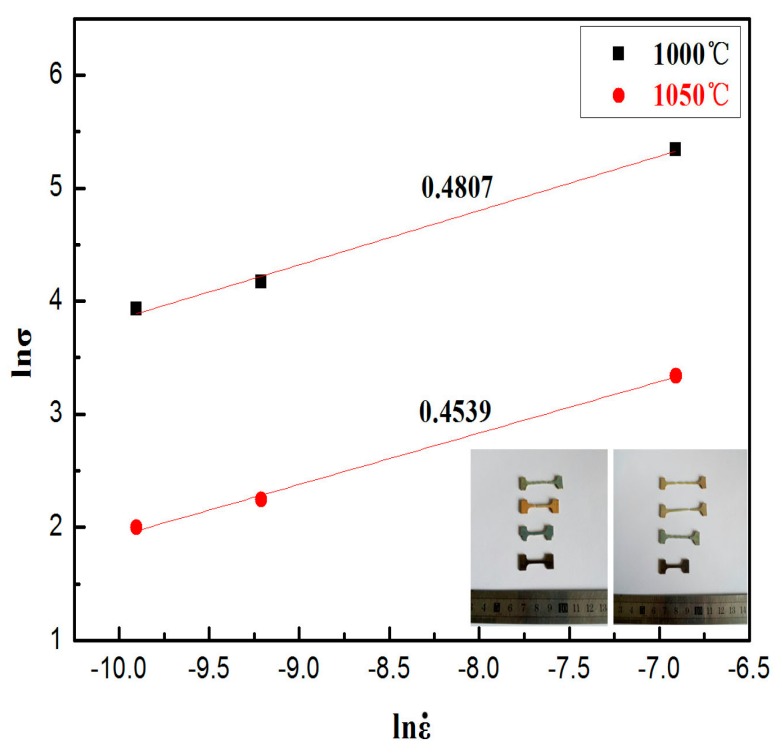
Determination of strain rate sensitivity index m according to the slope of ln*σ*-ln$\dot{\varepsilon }$.

The strain-rate sensitivity index *m* indicates the resistance of necking during superplastic deformation. Generally, a value of 0.3 for *m* is an indication of tensile superplasticity [22]. As shown in Figure 5, the *m* value of the alloy is measured to be 0.48 and 0.45 at 1000 °C and 1050 °C by the slope of ln*σ*-ln$\dot{\varepsilon }$ which indicates that the alloy exhibits superplasticity.

### 3.3. Analysis of the Fracture Surface

**Figure 6:**
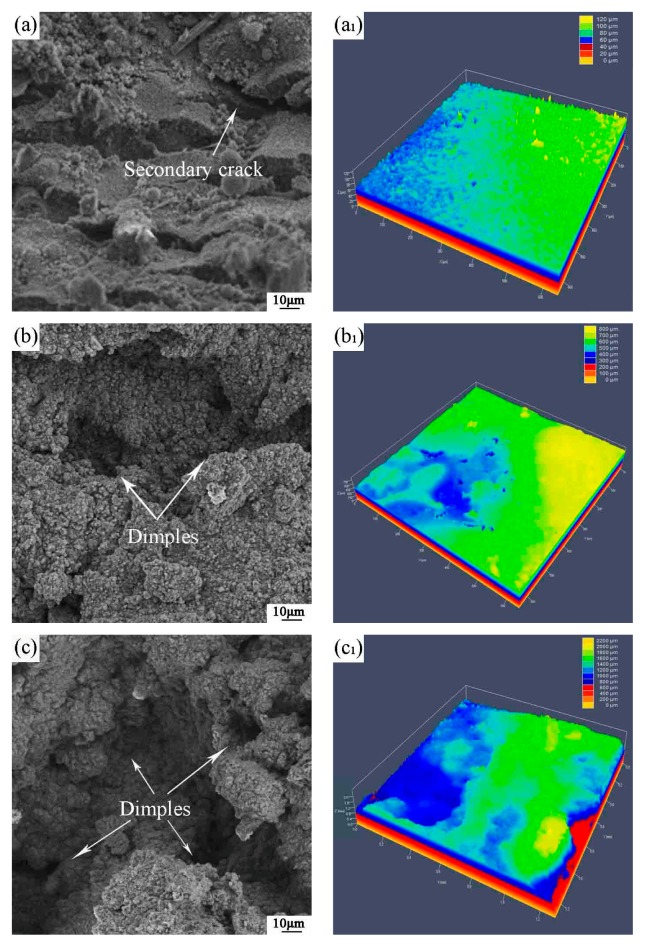
The tensile fracture at 1000 °C: (**a**) SEM micrographs at 1 × 10^−3^ s^−1^; (**a_1_**) LSM micrographs at 1 × 10^−3^ s^−1^; (**b**) SEM micrographs at 1 × 10^−4^ s^−1^; (**b_1_**) LSM micrographs at 1 × 10^−4^ s^−1^; (**c**) SEM micrographs at 5 × 10^−5^ s^−1^; (**c_1_**) LSM micrographs at 5 × 10^−5^ s^−1^.

The fractured surface has some secondary cracks (indicated by the arrow in Figure 6a). During the high-temperature tensile process, the initial cleavage cracks are hindered by grain boundaries, causing a large stress concentration near the grain boundary. Microcracks nucleate to expand and connect at the grain boundaries, which eventually leads to the fracture of the specimen. Meanwhile, there are a small amount of dimples in this condition and the depth of the dimples is 60 μm (as shown in Figure 6a_1_). As the strain rate is reduced to 1 × 10^−4^ s^−1^ and 5 × 10^−5^ s^−1^, the emergence of dimples can be observed at the fracture, revealing the occurrence of plastic deformation in the microstructures (indicated by the arrow in Figure 6b,c). With the decrease of the strain rate, the dimples gradually become deeper, from 600 μm to 1600 μm, and the number increases accordingly (as shown in Figure 6b_1_,c_1_). The resistance to plastic deformation is reduced, increasing elongation to exhibit superplasticity. Temperature has a significant influence on plastic deformation. High temperature lowers the elastic moduli which, in turn, promotes easy dislocation formation and glide. Therefore, the material becomes prone to plastic deformation. In the high-temperature tensile process, grain boundary sliding occurs more readily for superfine grains. The migration of atoms plays a regulatory role in the grain boundary slip. Due to the high mobility of grain boundaries, the grains gradually twist and move along the tensile direction. When the stress reaches its critical value, the alloy is fractured. The nature of fracture surfaces is a ductile fracture.

**Figure 7:**
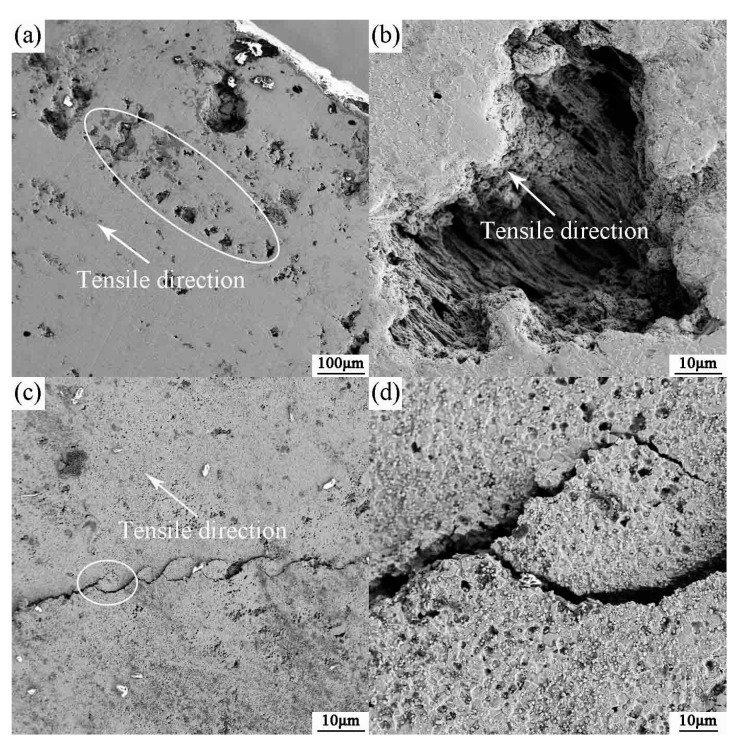
The SEM micrographs of tensile fracture at 1000 °C, 5 × 10^−5^ s^−1^ (**a**) the tensile fracture; (**b**) the magnified microcavities in (a); (**c**) near the tensile fracture; and (**d**) the magnified crack in (c).

Figure 7a illustrates the elongation of the grains along the tensile direction. The cavities shown are related to the damaged microstructures. It is observed that the cavities mainly appear at the grain boundaries leading to an inharmonious sliding of the grain boundaries. The magnified microcavities reveal the distribution of elongated microstructures along the tensile direction (as shown in Figure 7b). The growth of cavities is mainly controlled by plastic deformation, and so their size and number are larger at high temperature and under low strain rate conditions. However, both microstructure evolution and microcavity nucleation during the tensile process assist the sintered alloy to display superior tensile properties. In the initial stage of deformation, the formation of microcavities can promote the elongation in the tension tests, because the nucleation of cavities has a strong effect on reducing the concentration stress in local areas, especially in the grain boundaries, and this promotion continues to a critical strain [19]. Figure 7c shows a coherent microcrack in the SEM image of the microstructures near the tensile fracture and the microcrack develops at roughly 45° to the direction of the tensile force. As shown in Figure 7d, the enlargement of the circle reveals that the microcracks grow, expand, and connect along the grain boundary.

### 3.4. Deformation Mechanism

**Figure 8:**
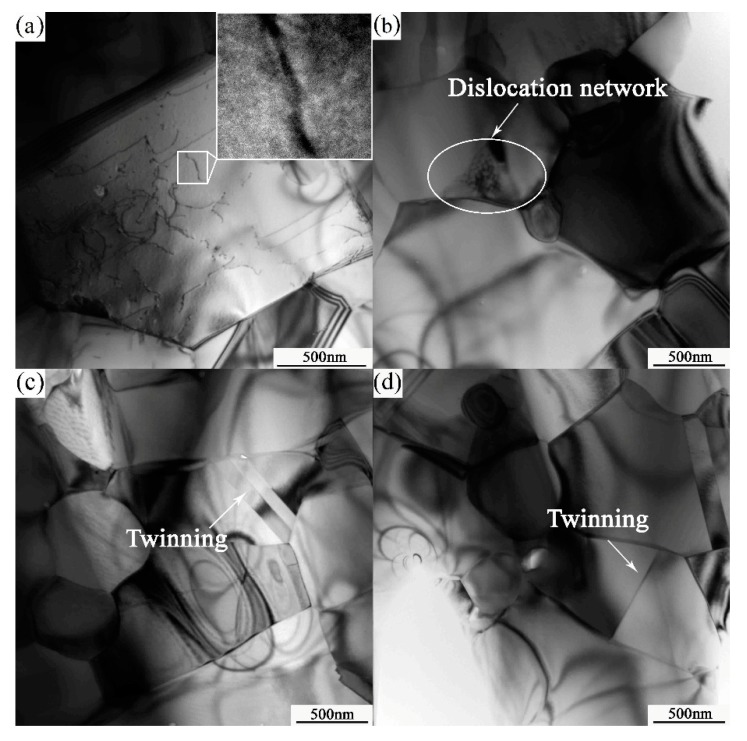
The TEM micrographs of tensile specimen at 1000 °C, 5 × 10^−5^ s^−1^ (**a**) dislocation network; (**b**) ordinary dislocation; (**c**) intra-granular twinning; and (**d**) interfacial twinning.

**Figure 9:**
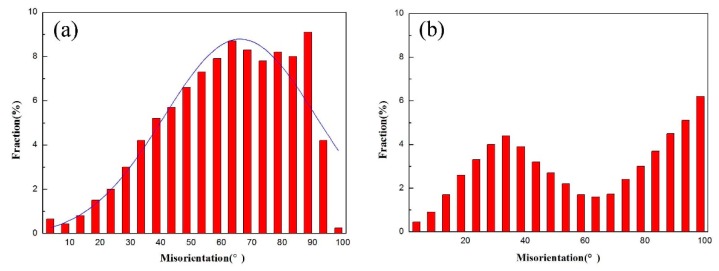
Histogram of the grain boundary misorientation of (**a**) sintered bulk; and (**b**) the deformation microstructure.

Figure 8 illustrates the TEM micrographs of the tensile specimen at 1000 °C and 5 × 10^−5^ s^−1^. The PM-TiAl-based alloy undergoes superplastic deformation, whereas the fine equiaxed grains are elongated. Meanwhile, it can be proved by the histogram of the grain boundary of the sintered bulk and deformation microstructure (as shown in Figure 9) that when the grain boundary slides, the sliding will cause stress concentration in its slip path and its vicinity. In order to coordinate the grain boundary slip, it is necessary to relax the deformation stress by dislocation movement. Therefore, a high dislocation density is observed near the grain boundary of the γ phase, and the dislocation type is the ordinary dislocation of the Burgers vector b = 1/2<110] (as shown in Figure 8a). The dislocation network is formed by the constant climbing and entanglement of the dislocations (as shown in Figure 8b). When the deformation conditions are favorable for superplasticity, that is, at a higher deformation temperature and lower strain rate (1000 °C, 5 × 10^−5^ s^−1^), the dislocation activity at grain boundaries is improved. The grain boundary is activated by the interaction of dislocations. Therefore, the grain boundary is shifted from the equilibrium state to a non-equilibrium state, improving the grain boundary migration. Thereby, grain boundary sliding and grain rotation are carried out, and deformation occurs along the most easily sliding surface.

In the elongated γ phase, the deformation twinning can be observed as the dislocations increase. The observed intra-granular twinning and interfacial twinning of the γ phase are shown in Figure 8c,d, respectively. According to TEM morphology, the twinning divides the γ/γ grains and the twin boundaries at the γ/γ interface are clear and distinct. Therefore, the high-temperature deformation of γ-TiAl-based alloy is the result of dislocation and twinning. Slip generally proceeds by four common unit dislocations of 1/2<110>{111}, eight superlattice dislocations of <101>{111}, and four twinning systems 1/6<112>{111} [23]. The dislocation and twinning are the coordination mechanism of grain boundary slip. The high mobility of the grain boundary must be responsible for superplasticity.

The mechanical properties of PM-TiAl-based alloys greatly depend on the interface structure, especially the grain boundary structure or the interface of fine grains. In this study, the phase interface after superplastic deformation was studied by means of the high-resolution structure.

**Figure 10:**
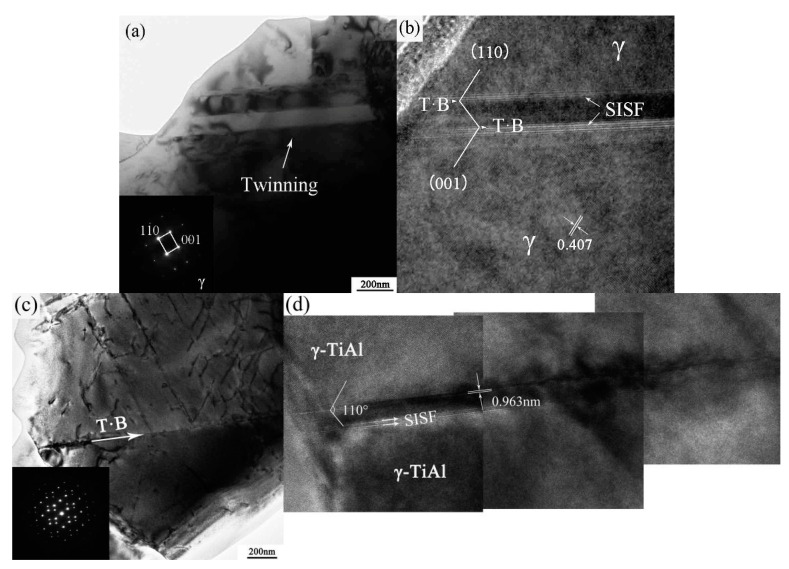
The HREM micrographs of superplastic deformation at 1000 °C, 5 × 10^−5^ s^−1^ of (**a**) intragranular twinning; and (**b**) interfacial twinning; (**c**) the direction of interfacial twinning; (**d**) interfacial twinning of the γ phase.

Figure 10 shows the HREM micrographs of superplastic deformation at 1000 °C and 5 × 10^−5^ s^−1^. The diffraction pattern analysis shows that the elongated grain is the γ phase. Figure 10b shows the intra-granular twinning of the γ phase at an angle of 110°. The twins on both sides of the grain boundary maintain a complete ordered structure. At the twin boundary, the two grains remain completely coherent. The orientation relationship between the γ/γ phase interface is (001)_γ_//(110)_γ_. The nucleation and propagation of the deformation twins in TiAl alloy are eventually attributed to the formation of a/6<112] Shockley incomplete dislocations and subsequent slipping on the corresponding {111}_γ_ [24,25,26]. The 1/2<110] perfect dislocations can be broken down into the 1/3<110> Frank incomplete dislocation and the 1/6<112] Shockley incomplete dislocation, and the dislocation decomposition relationship is [27]:

1/2 [110]→1/3 [111] + *SISF* + 1/6 [112] (1)

The slipping of the 1/6 [112] Shockley incomplete dislocation on the continuous (111)_γ_ surface will lead to the nucleation and propagation of the deformation twins. Additionally, 1/3 [111] Frank dislocations leave a dislocation step on the γ/γ interface. It is observed that 1/6<112] Shockley dislocations lead to the superlattice intrinsic stacking fault (*SISF*) at the tip of the deformed twin from the HREM images.

Figure 10c shows the nucleation and expansion of interfacial twinning at the γ/γ grain boundaries in the alloy. The HREM image of the γ phase interfacial twinning is shown in Figure 10d. The width of the twin boundary is 0.963 nm, and the angle of the twin boundary is 110°. Since the deformed twins are nucleated at the γ/γ grain boundaries, they are not available for non-uniform nucleation of perfect dislocations and superlattice dislocations. Therefore, it represents the uniform nucleation mechanism of twinning. Meanwhile, there is a large number of steps consisting of *SISF* on the twin interface, which results in the deviation of the twin interface. Therefore, the Shockley incomplete dislocation that produces the interface twin is not nucleated at the same location, but there are many nucleation points along the interface twins.

**Figure 11:**
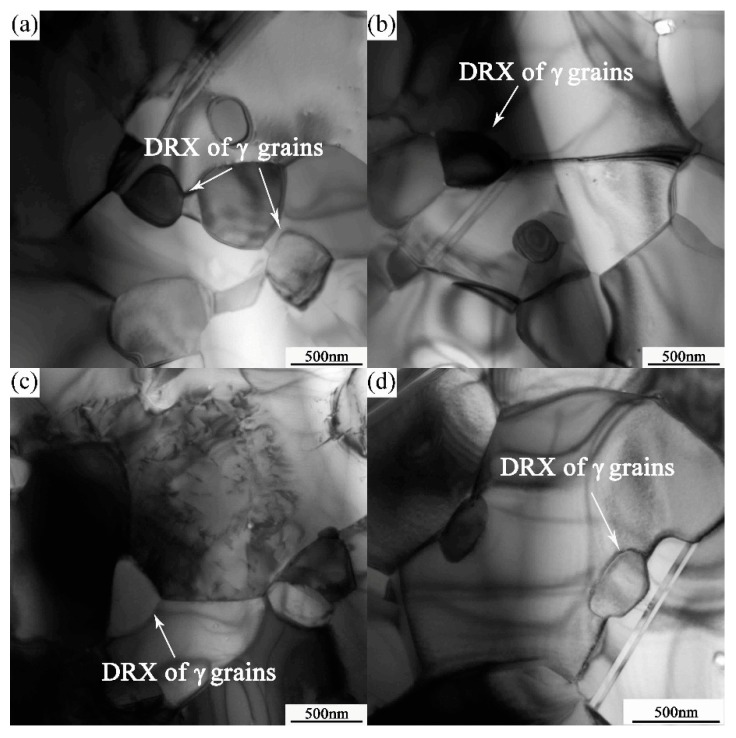
The TEM micrographs of dynamic recrystallization at 1000 °C, 5 × 10^−5^ s^−1^ (**a**) at the junctions; (**b**) at the grain boundaries; (**c**) near the dislocation; and (**d**) near the twinning.

**Figure 12:**
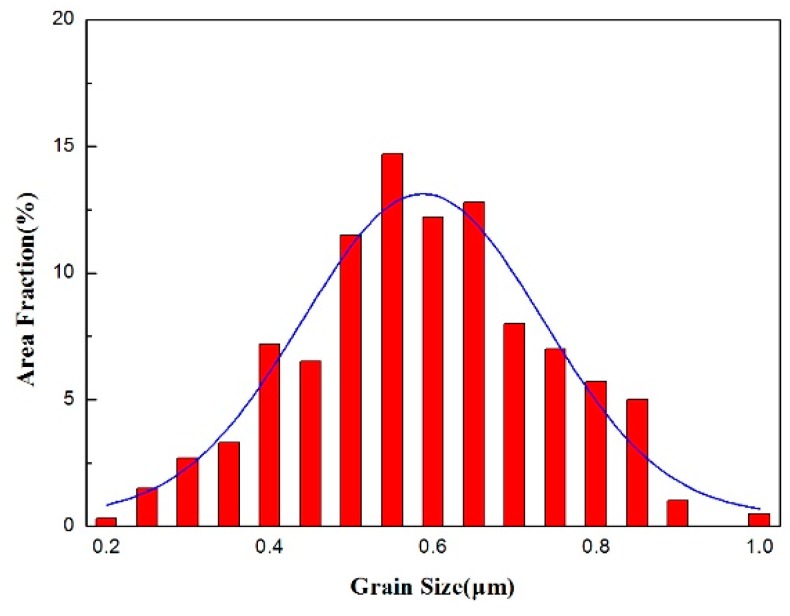
Histogram of γ grain size.

Dynamic recrystallization is an important structural feature observed for PM-TiAl-based alloys. The microstructure and phase distribution of the alloy can be more uniform while maintaining the stability of the structure. Unlike ordinary thermal deformation, the internal grain is no longer the nucleation position of dynamic recrystallization during superplastic deformation. The main nucleation positions of dynamic recrystallization occur mainly at the junctions and grain boundaries where the stress is highly concentrated, and near the dislocations and twins. As shown in the TEM micrographs of Figure 11 and Figure 12, the dynamic recrystallization occurring during superplastic deformation produces the average grain size is 275 nm and the fraction of recrystallized grains is 7.8%.

In the process of superplastic deformation, the deformation stress *σ* is proportional to the *a* power of the grain size *d* (0.7 < a < 2) [28]. Therefore, dynamic recrystallization can lead to grain refinement, especially at the junctions or at the triangular grain boundary, which makes the sliding of grain boundary easier, thus promoting the migration of grain boundary and the rotation of grains. Meanwhile, dynamic recrystallization is also an important stress relaxation mechanism. Therefore, the softening effect gradually dominates in the direction of the low hardening index, and changes to promote the reduction of flow stress.

## 4. Conclusions

1. The equiaxed superfine/nanocrystalline duplex (γ + α_2_) microstructure of the PM Ti-45Al-5Nb alloy has favourable superplasticity. The ultimate tensile strength of the alloy was 58.7 MPa at 1000 °C and 10.5 MPa at 1050 °C with a strain rate of 5 × 10^−5^ s^−1^, while the elongation was 121% and 233%, respectively.
2. The mechanism of superplastic deformation is dislocation slipping and twinning of the γ/γ phase. The orientation relationship between the γ/γ phase interface is (001)_γ_//(110)_γ_. The main softening mechanism is the dynamic recrystallization of γ grains. Dynamic recrystallization nucleates mainly at grain junctions and grain boundaries, where the stress is highly concentrated, and near dislocations and twins.
3. The plastic deformation mainly occurs in the matrix γ phase, and the dispersion of the equiaxed nanocrystalline α_2_ phase plays a promoting role. The α_2_ grains undergo rotation with the deformation of the γ phase in the process of superplastic deformation because of the fine grains and less slip of the system.

## Figures and Tables

**Table 1:** Comparison of various TiAl-based alloys parameters [18,19,20].

Composition (at %)	Grain Size (μm)	Tensile Temperature (°C)	Strain Rate (s^−1^)	Stress (MPa)	Elongation (%)	M
Ti-45Al-8Nb	10–20	1050	1 × 10^−3^	140	237	0.31
Ti-43.4Al-8Nb-0.2W-0.2B	4	1000	2.083 × 10^−4^	80	409	0.5
Ti-42.5Al-2.3Nb-2.2Cr-0.28W-0.15B	4	1000	1 × 10^−4^	90	450	-
Ti-45.2Al-3.5 (Nb,Cr,B)	3	1000	1 × 10^−3^	140	330	-
Ti-45Al-5Nb *	0.5–0.8	1050	5 × 10^−5^	10.6	233	0.45

* the nominal composition of this experimental alloy.
